# Prevalence and Correlates of Hand Dermatitis among Nurses in a Japanese Teaching Hospital

**DOI:** 10.2188/jea.13.157

**Published:** 2007-11-30

**Authors:** Derek Richard Smith, Kumeko Ohmura, Zentaro Yamagata

**Affiliations:** 1 Department of Health Sciences, Faculty of Medicine, University of Yamanashi.; 2 University Hospital, Faculty of Medicine, University of Yamanashi.

**Keywords:** hand dermatitis, nurse, Japan, epidemiology, risk factors

## Abstract

Background: Although hand dermatitis represents a common occupational disease among hospital nurses, epidemiologic studies of this nature are comparatively rare in Japan.

Methods: We recruited a complete cross-section of nurses from a teaching hospital in central Japan. Data was gathered by means of a self-reported questionnaire, with hand dermatitis symptoms and evaluation criteria drawn from previously validated research. Participants were categorised according to their hospital department during the analysis.

Results: A total of 305 questionnaires were successfully completed and returned (response rate: 84%). There were statistically significant differences in hand dermatitis prevalence between the departments (p<0.05), ranging from 6% in psychiatry to 48% in the surgical unit and averaging 35% across the entire group. A history of allergic disease was shown to increase the risk of hand dermatitis (odds ratio=3.7, 95% confidence interval: 2.1 - 6.6). Washing their hands more than 15 times per work shift also increased the risk (odds ratio=2.0, 95% confidence interval: 1.2 - 3.4).

Conclusion: This study has shown that hand dermatitis prevalence varies among Japanese nurses depending on their hospital department, and is generally quite high when compared to other reports.

Hand dermatitis (HD) represents a common occupational health problem of industrialized countries, and is usually a manifestation of irritant contact dermatitis or allergic contact dermatitis.^1^ Systemic allergy and atopic dermatitis are common endogenous predictors,^2^^-^^7^ while environmental influence represents an important exogenous risk factor in the development of this disease.^8^^,^^9^ Occupational exposure is particularly relevant, because many potential contact allergens, such as water and natural rubber latex, are often encountered during health care work.^4^^,^^5^ Hand washing frequency and place of employment are also known to be important risk factors.^8^^,^^9^ For these reasons, hospital workers are regularly affected by HD, with as many as 70% reporting the condition at least once in the previous year.^6^ Among them, nurses are known to be at particularly high risk. Various studies have shown the prevalence of nurses’ HD to be higher than for other job descriptions and the general community.^10^^,^^11^ Although the epidemiology of HD has been previously studied in other countries, similar research appears to be less common in Japan, and the prevalence among hospital nurses is generally unclear.

Self-reported surveys are a convenient and cost-effective tool for gathering information on hand dermatitis. Their validity and accuracy when compared to dermatologic examination for the same condition has been previously established.^12^^,^^13^ Furthermore, a study by Simion et al. demonstrated how self-reported information may also yield additional information on skin disease, particularly when the condition is below a clinically detectable level.^14^ Given the accuracy of dermatologic questionnaires and paucity of HD prevalence data, it was considered appropriate to investigate this occupational disease among hospital nurses in a selected area of central Japan using a self-reported survey. Because environmental exposure is known to influence HD prevalence,^8^ we also considered it necessary to stratify the nurses by work department and thus investigate environmental exposure as a risk factor.

## METHODS

This study involved a retrospective epidemiologic analysis of data gathered by means of a self-reported HD questionnaire. Our questionnaire was initially written in English, before being translated into Japanese and evaluated by a panel of bi-lingual researchers. It was then assessed for clarity within a Japanese occupational setting, before being back-translated into English and checked by the initial author. The instrument was a simple, two-page anonymous form including questions of age, department, weekly working hours, duration of employment, exposure to latex products, and the presence of systemic allergic diseases such as atopic dermatitis, asthma, allergic rhinitis, and hay-fever. Questions regarding the number of hand washes per work-shift and the presence of various HD symptoms were also asked.

All HD symptom descriptions were adapted from previous questionnaire-based investigations.^8^^-^^11^ Briefly, they included red and swollen hands or fingers, red hands and fingers or fissures, vesicles on the hands or between the fingers, scaling hands or fingers with fissures, and itching hands or fingers with fissures.^10^^,^^11^ A detailed description of HD symptoms is shown in the appendix. Nurses were asked whether any of these symptoms had occurred in the previous 12 months, and if so, had any case persisted for longer than 3 weeks or reoccurred during that time. A positive case was defined using previously validated criteria (2 or more symptoms appearing in the last 12 months and persisting for more than 3 weeks or reoccurring during that time).^10^^-^^12^ Upon receiving their questionnaire at work, nurses within the selected departments were asked to return their surveys within one week. All responses were collected during the late spring / early autumn season in 2002.

Data was entered into a common spreadsheet program before being analysed by JMP^®^ statistical software (JMP Version 4: SAS Institute, 2001). Descriptive statistics and prevalence were analyzed by department of employment. Significant differences between the departments were calculated using the chi-square test for discrete variables and one-way ANOVA for continuous variables. Logistic regression was also performed to determine HD correlations with work environment and endogenous factors. During regression analyses, the presence of hand dermatitis was utilised as the dependent variable with various demographic and workplace factors selected as the independent variables. Risk factors were initially evaluated as crude odds ratios (OR) with 95% confidence intervals (95% CI). To avoid confounding, values were then adjusted for variables with statistically significant differences between the departments (age, alcohol consumption, latex glove usage, and total duration of employment). HD risk factors identified during previous studies (systemic allergy, number of hand washes per shift and department of employment) were also added to the regression models. Because handwashing frequency was not normally distributed, we used the median number of hand washes per shift as the cut-off threshold and evaluated it as a discrete variable. All risk factors were then analyzed simultaneously using multiple logistic regression models and expressed as adjusted odds ratios. Probability values (p) above 0.05 were regarded as statistically insignificant throughout the analyses.

## RESULTS

We recruited a complete cross-section of 363 registered nurses from a university hospital in Yamanashi prefecture, central Japan. The response rate ranged from 74% (Internal Medicine) to 100% (Psychiatry), with an overall rate of 84% across the departments. Two questionnaires were initially excluded for incompleteness, with 4 males also excluded to reduce gender confounding; leaving a total of 305 female, registered nurses. Their average age varied between the departments (p<0.0001), ranging from 27.4 years to 34.9 years (Table 1). Alcohol consumption also varied (37% to 73%, p<0.05), while tobacco smoking was reported by between 13% and 27% of them. The prevalence of systemic allergy was also reasonably high (19% to 71%), as was latex glove usage (31% to 82%, p<0.01). Nurses’ total length of employment varied between the departments (6.0 years to 11.0 years, p<0.05), as did their average number of hand washes per shift (13.6 to 20.9 hand washes per shift, overall mean: 18.1 ± 10.7 hand washes per shift). The median number of hand washes per work shift was 15. The prevalence of self-reported hand dermatitis by department ranged from 6% to 48%, with an average prevalence of 35% across the entire group. This variation was statistically significant (p<0.05). Other skin diseases were also self-reported by nurses during our study, with an average prevalence as follows: acne (32%), atopic dermatitis (12%), tinea pedis (3%), and tinea unguium (1%).

**Table 1.** Nurse characteristics and self-reported hand dermatitis prevalence by department.

	Surgery			Intensive care			Internal medicine			General			Obstetrics/ gynaecology			Psychiatry		

	n		(%) ^a^	n		(%) ^a^	n		(%) ^a^	n		(%) ^a^	n		(%) ^a^	n		(%) ^a^
Demographic ^b^																		

Age (years)***	27.9	±	6.2	27.4	±	5.5	28.5	±	6.1	34.9	±	10.2	29.4	±	7.6	28.5	±	7.7
Alcohol*	50		(65)	42		(63)	34		(53)	15		(37)	29		(73)	10		(63)
Tobacco	19		(25)	18		(27)	16		(25)	8		(20)	6		(15)	2		(13)
Allergy	55		(71)	21		(31)	21		(33)	13		(32)	16		(40)	3		(19)
Workplace ^b^																		

Latex glove use**	33		(43)	55		(82)	41		(64)	21		(51)	31		(78)	5		(31)
Weekly hours	52.2	±	53.7	49.6	±	46.5	49.4	±	11.2	44.9	±	10.5	45.8	±	5.8	42.0	±	6.3
Total job years*	6.4	±	6.2	6.0	±	6.5	6.7	±	6.1	11.0	±	9.8	7.4	±	6.8	6.5	±	7.3
Hand washes ^c^	18.4	±	10.9	18.9	±	14.6	16.4	±	8.4	18.0	±	9.6	20.9	±	9.5	13.6	±	5.7

Hand dermatitis*	37		(48)	24		(36)	20		(31)	15		(37)	11		(28)	1		(6)

^a^ Percentages of each department are shown in parenthesis (n=77, 67, 64, 41, 40 and, 16 respectively).

^b^ Significant differences between the departments were calculated using the chi-square test for discrete variables and one-way ANOVA for continuous variables (*:p<0.05, **:p<0.01, and ***:p<0.0001).

^c^ Average number of hand washes per shift.

Three HD risk factors were identified during regression analysis, two of which remained statistically significant after adjustment for confounding variables (Table 2). A previous history of allergic disease was shown to increase HD risk 3.7 times among the nurses within this study. The risk of HD also increased linearly in proportion to hand washing frequency when assessed as a continuous variable (p<0.01). However, as the median number of hand washes for the entire group was 15, we chose 15 as an appropriate cut-off point during regression analysis. Washing their hands more than 15 times per work shift was shown to increase the risk of HD 2-fold. Working in the surgical department also increased the risk of HD 1.8 times when assessed as a crude variable, however, this effect disappeared after adjustment for age, alcohol consumption, latex glove usage, total duration of employment, the presence of systemic allergic disease, and the number of hand washes per work shift.

**Table 2.** Risk factors associated with self-reported hand dermatitis among hospital nurses

Risk factor	Category n(%) ^a^			Crude odds ratio ^b^		Adjusted odds ratio ^c^	
				(95% confidence interval)		(95% confidence interval)	
Allergy ^d^	No	249	(82)	1.0	-	1.0	-
	Yes	56	(18)	3.4	(2.0 – 5.8)	3.7	(2.1 – 6.6)
Hand washes ^e^	<15 / shift	168	(55)	1.0	-	1.0	-
	>15 / shift	137	(45)	2.1	(1.3 – 3.4)	2.0	(1.2 – 3.4)
Department	Other	268	(88)	1.0	-	1.0	-
	Surgery	37	(12)	1.8	(1.1 – 3.2)	1.8	(1.0 – 3.2)

^a^ Percentage of all nurses in each subcategory (n=305).

^b^ Risk factors calculated using simple logistic regression and expressed as crude odds ratios with 95% confidence intervals.

^c^ Risk factors analysed simultaneously using multiple logistic regression and adjusted for age, alcohol consumption, latex glove usage, total duration of employment, presence of systemic allergic disease, number of hand washes and working in the surgery department.

^d^ Refers to systemic allergic disease such as atopy, asthma, hay-fever or allergic rhinitis.

^e^ The median number of hand washes per shift was 15 times

## DISCUSSION

With an overall prevalence of 35%, the hospital nurses within this study reported more HD than previously documented among nurses in the United States (25.9%),^8^ and in the Netherlands (between 29.4% and 32.0%).^11^ The proportion was also higher than community investigations undertaken in Sweden (11.8%)^17^ and the Netherlands (10.0% and 10.6%).^18^^,^^11^ The general population prevalence of HD has not yet been established in Japan, but a previous study revealed this disease among 23.8% of Japanese nursing students.^16^ Dutch nursing students, however, seem to have a lower incidence than their Japanese counterparts (13.5%).^7^ When compared to other hospital employees, our Japanese nurses reported HD at a level somewhere between that suffered by Italian (21.2%),^19^ American (55.6%)^9^ and Polish (69.6%)^6^ hospital staff. Alternatively, the prevalence of miscellaneous skin diseases such as atopy, acne, and tinea pedis was reasonably consistent with previous community studies.^20^^,^^21^

The reasons for these differences in HD prevalence are difficult to ascertain, although there are a few plausible explanations. As the current study was conducted at the end of spring, it is feasible that our elevated prevalence reflects seasonal atopic dermatitis increases occurring during springtime in Japan.^16^ Seasonal influence is a particularly attractive hypothesis as atopy was both highly prevalent and significantly correlated with HD. Given this potential confounding variable, it would be interesting to repeat the study in a different season and thus investigate seasonal effects on HD prevalence. Some other possibilities are also worth considering. Because our study was based on a self-reported questionnaire, it was implicitly dependent on self-assessment and self-diagnosis. Certain differences may have occurred between our current results and other studies where conditions were predominately physician-diagnosed. Nevertheless, as educated health professionals, registered nurses probably understand HD symptoms reasonably well and are therefore capable of a fairly accurate self-diagnosis.

A psychological selection effect may have occurred where people who suffer from chronic diseases (like atopic skin disease) feel some degree of empathy for patients, and thus gravitate towards employment with health care professions such as nursing. This situation might result in a cohort markedly different from the general population, and one whose members suffer unusually high rates of skin disease such as HD. Similarly, differences in HD prevalence may have arisen from slight variations in detection methodology between the previously mentioned investigations. Intrinsic cultural differences may also play a part in disease reporting behaviour between Japanese nurses and their counterparts in other countries.

The risk factors identified by logistic regression during our study are all consistent with previous reports. A history of allergic disease was found to be the most important predictive factor in HD development. Even after adjustment for confounding variables, nurses with allergy were 3.7 times more likely to report HD. This result supports previous research implicating allergy as a HD risk factor among both hospital nurses and hospital workers in general.^2^^,^^5^^,^^6^^,^^19^ Atopic skin is believed to be more easily irritated than in non-atopics, while skin repair is generally prolonged with frequent relapses. Acute HD symptoms may also be more severe among atopies.^9^ These mechanisms go a long way in explaining the prolonged nature of HD among hospital nurses with pre-existing allergic disease. Interestingly, however, latex glove usage was not statistically related to HD during the current study, despite its known properties as both a contact allergen within health care settings^1^^,^^19^ and a correlate for HD.^8^ There are a few possible reasons for our lack of association with latex gloves when compared to other research. Firstly, there is the issue of sample size. Two previous studies of hospital nurses’ HD utilised a convenience sample^8^ and a much smaller sample size,^9^ both of which may have biased their overall results. Secondly, our definition of hand dermatitis required the presence of at least 2 physical symptoms and either a recurring or protracted course.^10^^,^^11^ As such, latex glove usage might be a genuine risk factor for minor skin damage of the hands, but less important when the damage is severe or protracted.

Hand washing frequency was shown to be the second most important risk factor, increasing the odds of reporting HD 2-fold among nurses who washed their hands more than 15 times per work shift. Hand washing has previously been demonstrated as a significant risk factor for HD among hospital nurses.^8^^,^^9^ Similarly, wet-work of any description represents another predictive variable common amongst hospital employees.^2^^,^^5^ Because handwashing frequency was not normally distributed, we used the median number of hand washes per shift as the cut-off threshold when investigating this particular risk factor. Our methodology differed slightly from that employed by Larson et al.,^8^ who also evaluated hand washing frequency as a HD risk factor among American hospital nurses. In their study, a significant statistical association between the two factors was found, although their average hand washing frequency (ranging from 25.7 to 34.5 washes per shift) was much higher than ours (18.1 hand washes per shift). Similarly, Forrester et al.^9^ investigated HD risk factors and showed correlations with hand washing frequency, again with a higher cut-off point than ours (35 washes per shift). Although there were some slight differences between our research methodology and that of others, these results indicate that handwashing remains an important correlate of HD among hospital nurses. Moreover, our study confirms that increasing hand-wash frequency is probably the most important component of this risk.

As the prevalence of HD varied significantly between the departments, we also conducted regression analyses with respect to work environment. Employment in the surgical department was found to be a HD risk factor by the crude analysis and was also independent of age, work duration, latex glove usage, allergy, and hand washing frequency. However, the effect diminished when other risk factors were added to the regression model. Nonetheless, this result suggests the existence of some HD risk factor intrinsic to the surgical department. The most likely candidate would appear to be disinfectant solutions, many of which are routinely encountered during surgical work. Stingeni et al.^19^ reported disinfectants as the most common cause of occupational hand dermatitis within an Italian hospital. Similarly, Larson et al.^8^ also demonstrated that disinfectant category was correlated with HD development among hospital nurses. From these results, we surmise that environmental exposure to disinfectant solution probably represents another important risk factor in the development of HD among Japanese nurses as throughout the world.

## CONCLUSION

Although this investigation suffered certain limitations, HD among a cross-section of Japanese nurses has been documented for what appears to be the first time. We have shown that HD prevalence varies between the departments and is probably related to intrinsic environmental exposure therein. Higher HD proportion among our group also suggests that Japanese nurses may be more susceptible to the irritant effects of disinfectant solutions than their counterparts in other countries. Further research is suggested to elucidate the aetiology of nurses’ HD and test these preliminary hypotheses however.

## Appendix.

Questions on hand dermatitis symptoms (translated into Japanese) ^a^
